# On Sequence Learning Models: Open-loop Control Not Strictly Guided by Hick’s Law

**DOI:** 10.1038/srep23018

**Published:** 2016-03-15

**Authors:** Rodrigo Pavão, Joice P. Savietto, João R. Sato, Gilberto F. Xavier, André F. Helene

**Affiliations:** 1 Universidade Federal do Rio Grande do Norte, Instituto do Cérebro, Natal, 59056–450, Brazil; 2 Universidade de São Paulo, Instituto de Biociências, São Paulo, 05508-090, Brazil; 3 Universidade Federal do ABC, Centro de Matemática, Computação e Cognição, Santo André, 09210-580, Brazil

## Abstract

According to the Hick’s law, reaction times increase linearly with the uncertainty of target stimuli. We tested the generality of this law by measuring reaction times in a human sequence learning protocol involving serial target locations which differed in transition probability and global entropy. Our results showed that sigmoid functions better describe the relationship between reaction times and uncertainty when compared to linear functions. Sequence predictability was estimated by distinct statistical predictors: conditional probability, conditional entropy, joint probability and joint entropy measures. Conditional predictors relate to closed-loop control models describing that performance is guided by on-line access to past sequence structure to predict next location. Differently, joint predictors relate to open-loop control models assuming global access of sequence structure, requiring no constant monitoring. We tested which of these predictors better describe performance on the sequence learning protocol. Results suggest that joint predictors are more accurate than conditional predictors to track performance. In conclusion, sequence learning is better described as an open-loop process which is not precisely predicted by Hick’s law.

Simple choices are performed faster than difficult ones. The classical study by Hick^1^ explored this intuitive notion which was later formalized as the Hick’s Law: “reaction time (RT) increases as a linear function of the log of the number of stimuli alternatives and thus, in information theory terms, RT is proportional to stimulus uncertainty”^2^. Hick’s pioneer study involved the choice reaction time protocol and manipulated uncertainty by varying the number of locations. We tested the generality of Hick’s Law in modified versions of the protocol created by Nissen and Bullemer^3^, named serial reaction time (SRT) task (Fig. 1a). The original SRT task comprised a repeating 10-trial stimulus sequence, therefore, including identifiable repetitive rules. Note that the number of locations is fixed and there are repetitive sequences in SRT task, which is not the case for the choice reaction time task employed by Hick^1^. Presently, different laboratories adopt SRT task to investigate sequence learning^4^,^5^,^6^,^7^,^8^,^9^,^10^,^11^. Because of the repetitive nature of the sequence and also the possibility of introducing probability controlled transitions between locations, SRT task suits to evaluate the effects of predictability on performance. In addition, performance gradually changes along training and changes in the sequence of locations may be introduced along with continuous performance of the task without prior warning. One of the aims of the present study was to evaluate whether Hick’s Law suits to a sequence learning protocol; i.e., if performance on SRT task follows a linear function with stimuli uncertainty.

Two different statistical predictors, which relate to distinctive assumptions about underlying cognitive processes, have been employed by distinct authors to describe RTs in SRT task. Sequence entropy (also referred as “joint entropy”) is a measure of uncertainty associated with a set of transitions of a sequence of locations^4^,^6^. Transition conditional probability^5^,^12^ is the probability of occurrence of a certain location given that some other locations have already occurred (check the explanation about the statistical predictors in Methods section). Conditional predictors relate to closed-loop models and assume that it occurs on-line access to the sequence structure for prediction of the next location^13^,^14^. In contrast, joint predictors relate to open-loop models and assume that there is a global access to sequence structure^15^ or motor schema responsible for execution of the sequence^16^. Moreover, closed-loop models involve constant monitoring of past locations to improve perception and/or action towards the likely future location, and thus requires high cognitive demand^13^,^14^. On the other hand, open-loop model requires no constant monitoring allowing performance under low cognitive demand^16^,^17^,^18^. And additional aim of the present study was to evaluate the plausibility of these cognitive models of sequence learning. We compared the explanatory power of the conditional and joint predictors to the actual RTs on SRT task involving sequences with distinctive and controlled statistical predictors.

The predictability of sequences performed in the SRT task was manipulated in order to test the two above described hypotheses, namely (1) linear versus non-linear function relating RTs and uncertainty, and (2) closed versus open-loop model controlling the sequence learning. The sequences performed ranged from extreme unpredictability (random sequence, which usually take longer RTs) and extreme predictability (repetitive sequence, which usually take short RTs). The data from two behavioral experiments involving SRT task in healthy human volunteers showed that RTs are better described by joint predictors than conditional predictors, thus supporting open-loop models rather than closed-loop models. In addition, data showed that both probability and entropy measures relate to RTs following a sigmoid function instead of a linear function, thus revealing that sequence learning in SRT tasks is not strictly governed by Hick’s Law. This conclusion was reaffirmed by description of RT dynamics along training blocks using sigmoid curve parameters.

## Results

### Reaction times across training blocks

This first set of analyses describes how performance is affected by repetitive training and by qualitative sequence uncertainty. Note that only data of Experiment 1 were evaluated and that uncertainty quantification is not required for these analyses.

**Figure 1:**
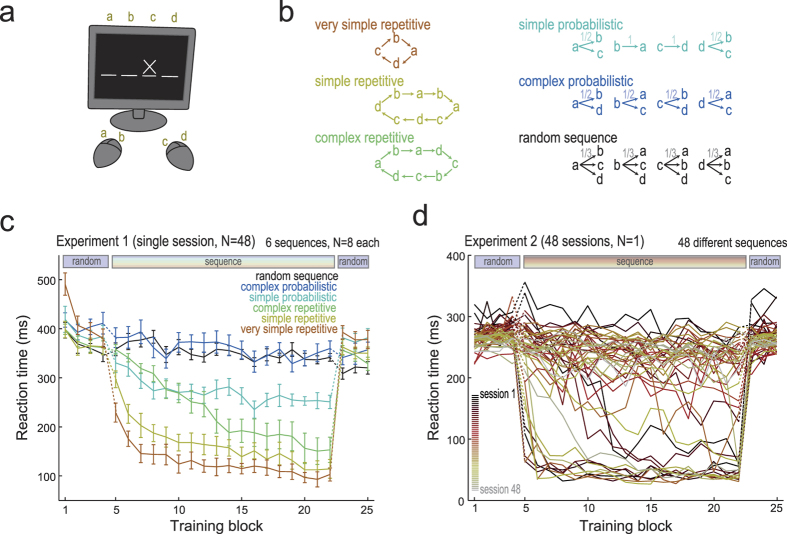
Experiment design and reaction times (RTs) across training blocks. (**a**) Serial reaction time (SRT) task apparatus. The experiment consisted of 1225 trials, divided in 25 blocks of 49 trials. In each trial the volunteer should press the button on the mouse devices corresponding to the stimulus location. (**b**) Each volunteer of Experiment 1 performed one of six sequence types displayed. The sequences could be either repetitive (deterministic) or probabilistic, shown on left or right, respectively. (**c**) Experiment 1: RTs across training blocks for each group of volunteers. All volunteers performed random sequences on blocks 1 to 4 and 23 to 25; from blocks 5 to 22 they performed one of the sequences presented in Fig. 1b. Results are denoted by the mean of the median RT for each volunteer in each training block; bars denote standard error of the mean (N = 8). Notice that the RTs increased with the complexity of the sequence. (**d**) Experiment 2: median RTs across training blocks for each session. Experimental design was the same of Experiment 1, except for (i) a wide variety of sequences was used (see Supplementary Table S1), and (ii) only one volunteer performed all the sequences in different sessions (one session per day). Notice that the RTs cannot be explained only by the session number.

The ANOVA involving RTs on blocks 1 to 4 of Experiment 1 (Fig. 1c) revealed lack of significant main Group effect (*F*_5,42_ = 1.62; *p* = 0.1762; 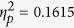) and significant main Block effect (*F*_3,126_ = 42.14; *p* < 0.0001; 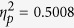). Bonferroni’s post-hoc (BPH) test revealed that block 1 differs from the other blocks. Additionally, there was significant Block x Group interaction effect (*F*_15,126_ = 3.80; *p* < 0.0001; 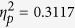). The BPH test revealed that one of the groups was slower as compared to the remaining groups on the first block. The groups were globally homogeneous and improved perceptual-motor ability along repetitive retraining with a random sequence.

ANOVA involving RTs on Blocks 5 to 22 of Experiment 1 (Fig. 1c) revealed a significant main Group effect (*F*_5,42_ = 42.56; p < 0.0001; 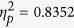). The BPH test revealed that all groups differed from each other, except random versus complex probabilistic, simple probabilistic versus complex repetitive and simple repetitive versus very simple repetitive. ANOVA also revealed a significant main Block effect (*F*_17,714_ = 48.30; *p* < 0.0001; 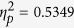). The BPH test revealed that early blocks differ from late blocks and that consecutive blocks did not differ among each other. Finally, ANOVA revealed a Group x Block interaction effect (*F*_85,714_ = 5.05; *p* < 0.0001; 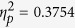). The BPH test replicated the effects observed for main factor effects of Group and Block, thus indicating that the predictability of the sequences strongly impacted rate of acquisition.

In fact, as Fig. 1c shows, acquisition of repetitive and thus more predictable sequences resulted in quicker decrements in RTs along training. In addition, shorter RTs were associated with less complex sequences (check performance for very simple repetitive, simple repetitive and complex repetitive sequences along blocks 5 to 22). In contrast, simple probabilistic sequences generated both intermediate acquisition rates and intermediate performance at asymptotic levels. Finally, complex probabilistic sequences and random sequences generated slower acquisition rates and higher RTs at asymptotic levels as compared to the other sequences.

Relative to RTs on blocks 23 to 25 of Experiment 1, when random sequences were presented for all groups, ANOVA revealed^1^ a significant main Group effect (*F*_5,42_ = 2.83; *p* = 0.0272; 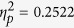) (random and very simple repetitive groups differed from each other according to the BPH test)^2^, a Group x Block interaction effect (*F*_10,84_ = 2.28; *p* = 0.0207; 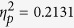) (random and very simple repetitive groups differed from each other, only on block 1, according to the BPH test), and^3^ lack of significant main Block effect (*F*_2,84_ = 1.02; *p* = 0.3668; 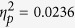). Since random sequences were employed for all subjects along blocks 23 to 25, group differences are not ascribable to the present structure of the sequence. Inspection of Fig. 1c shows that RTs on blocks 23 to 25 follow an inverse relationship with the predictability of the sequences to which the subjects had been exposed during prior training on blocks 5 to 22, thus suggesting an interference of training with the previously acquired sequence on performance of the random sequence.

### Reaction times and probability measures

**Figure 2:**
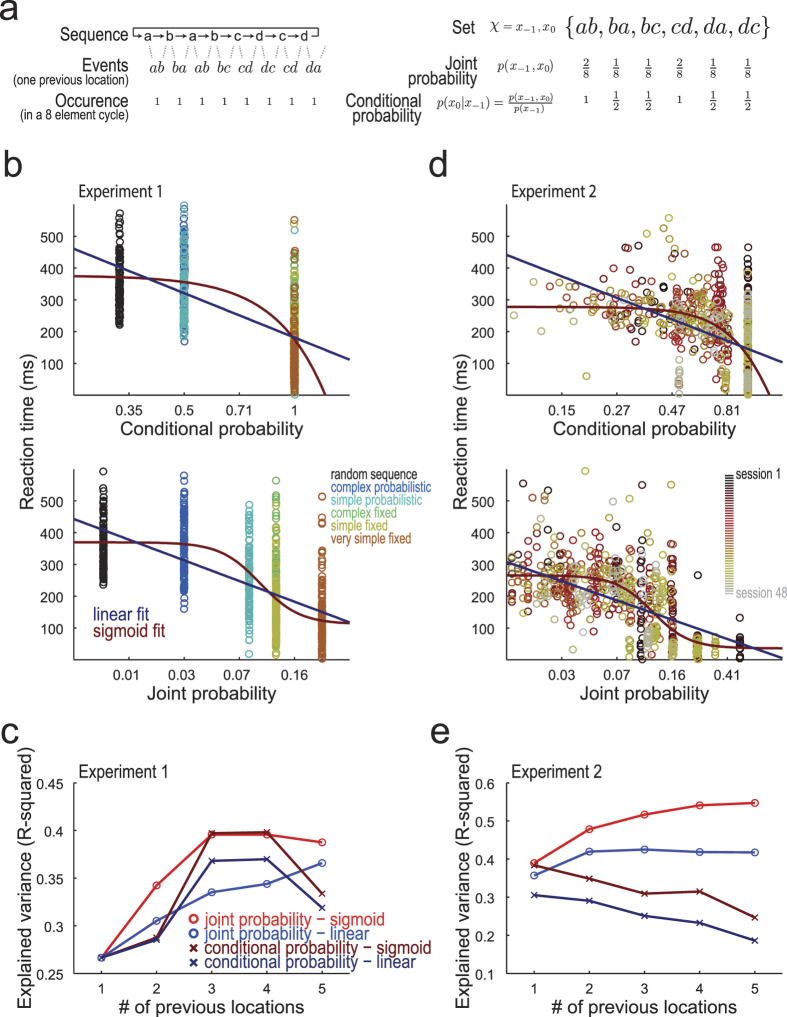
RTs are best predicted by joint probability following sigmoid function. (**a**) Calculation of joint probability (JP) and conditional probability (CP). From the sequence performed by the volunteer, we built a set of transitions which represents the locations along present trials (X0) as well as the locations of one previous trials (X − 1). Moreover, additional previous locations (X − 2, X − 3, etc) could also be included in the constitution of the set. The probabilities were calculated from the relative frequency the transitions of the set. (**b**) RT as a function of CP and JP for Experiment 1. Sigmoid and linear functions were fit to the raw RT vs. probability distributions. The color of the data point indicate the sequence performed by the volunteers. The transition probabilities were calculated considering three previous elements. (**c**) Explained RT variance by JP and CP, using linear and sigmoid function in Experiment 1. The JP associated to sigmoid function explain RTs variance equally or better than the other combinations for each number of previous locations used to constitute the transitions. (**d**) Same as Fig. 2b, for Experiment 2. The data point color indicate the number of the session. (**e**) Same as Fig. 2c, for Experiment 2. The RTs were best described by the sigmoid function and the JP values.

We evaluated how the RTs relate to probability (Fig. 2). Figure 2b shows the dispersion between RTs and conditional probability (CP; top panel) and joint probability (JP; bottom panel) for Experiment 1. In both panels, transitions of higher probability tend to be performed faster than transitions of low probability. The sigmoid function describe the average relationships slightly better than the linear function. We compared the linear and sigmoid functions and the CP and JP measures on their competence to describe RTs with the R-squared measure of goodness of fit (Fig. 2c). The sigmoid function predicted RTs better than linear function. The JP tends to be a more powerful predictor, but its performance ties with CP when three or four previous locations constitute the set of transitions.

Each data point of the dispersion plots in Fig. 2b refers to one single trial of one single volunteer. The curves were fit to pooled observations from trial 442 to 1078 from all volunteers, thus avoiding the fast-changing RT over the first blocks of training (check Fig. 1c). Additionally, we displayed only 2.5% of the total of data points to avoid overcrowd and ease the visual inspection. The X-axis of Fig. 2c indicates the number of previous locations constituting the transitions which had their probabilities calculated: when two previous locations are considered, together with the present locations, the set of transitions was constituted by three locations. The X-axis values of the dispersion plots of Fig. 2b are probabilities of transitions constituted with three previous locations; the following analysis used the same number of previous locations.

Figures 2d,e show similar analyses for data of Experiment 2. For this wide range of dense probability values, the RTs are beyond question better predicted by JP under a sigmoid function. It shows that about 50% of the variance of the noisy distribution of single-trial RTs is explained by JP and sigmoid function. The quantitative difference in the R-squared analysis between the experiments (Fig. 2c,e) is likely ascribable to the lower variety of sequences and higher heterogeneity of the volunteers in Experiment 1.

### Reaction times and entropy measures

**Figure 3:**
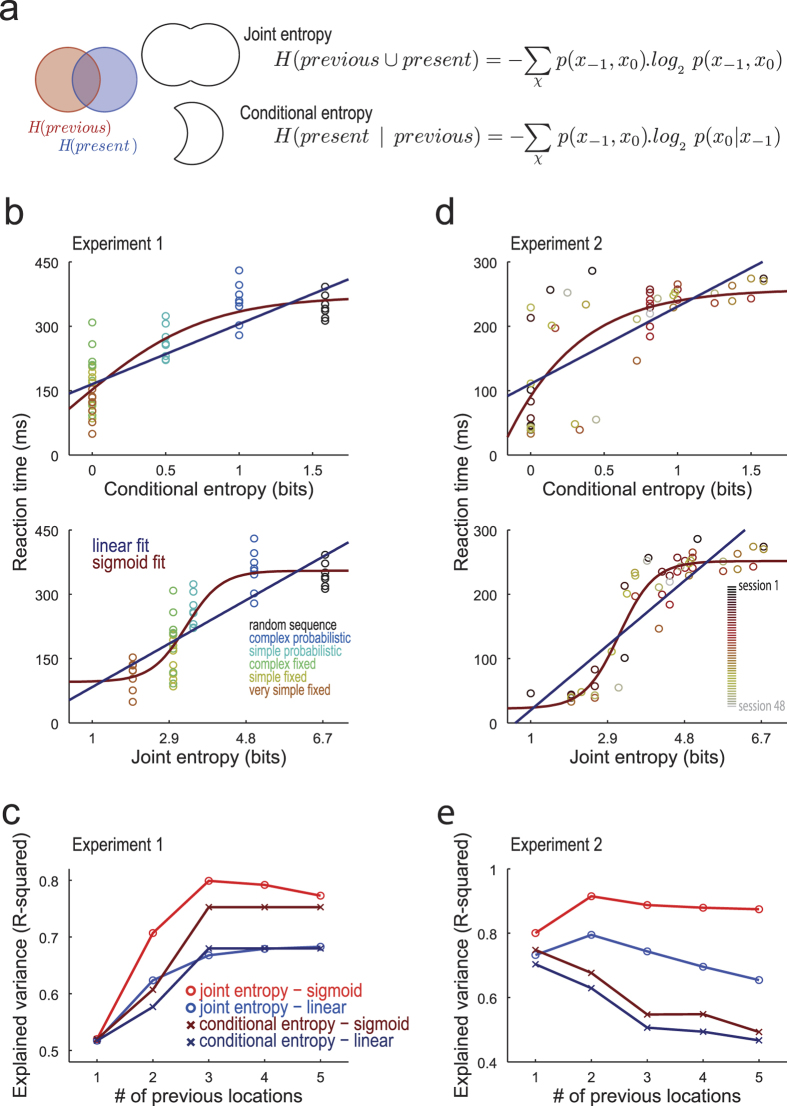
Median RTs of the sequences are best predicted by joint entropy following sigmoid function. (**a**) Calculation of joint entropy (JE) and conditional entropy (CE). The equations describe how the entropy values can be calculated directly from the joint and conditional probability (described in Fig. 2a). The JE is the entropy of the set of transitions representing previous and present locations, while the CE is the entropy of previous locations subtracted from the JE (see diagram in the left of the panel). (**b**) RTs as a function of CE and JE for Experiment 1. Each data point indicates the sequence entropy and the median RT of each subject of Experiment 1. (**c**) Explained median RTs variance by JE and CE, under sigmoid and linear fit, for Experiment 1. The variance of median RTs was best or equally well described by the sigmoid fit on the JE values, for each number of previous locations used to constitute the transitions. (**d**) Same as Fig. 3b, for Experiment 2. Data points indicate the sequence entropy and the median RTs of each session. (**e**) Same as Fig. 3c, for Experiment 2. Again, JE values under a sigmoid fit explains maximally the variance of median RTs.

A description on how the session averaged RTs relate to the entropy measures is presented on Fig. 3. Each sequence structure was summarized in one value of conditional entropy (CE) and one value of joint entropy (JE). Figure 3b shows the dispersion of the median RTs of each subject and the CE (top panel) or JE (bottom panel) for Experiment 1. Both measures reveal that low entropy sequences are performed faster than high entropy sequences. Linear and sigmoid functions describe RTs similarly using CE measures (Fig. 3b, top); however, for JE , sigmoid function is much superior to linear function (Fig. 3b, bottom). The systematic comparison presented in Fig. 3c show that sigmoid function and JE predictors are clearly the best combination for describing RTs. Moreover, Experiment 2 data analyses (Fig. 3d,e) revealed the same finding, with an impressive 90% of variance of median RTs time explained by sigmoid function and JE.

### Changes on the sigmoid fit on the joint entropy values across training blocks

**Figure 4:**
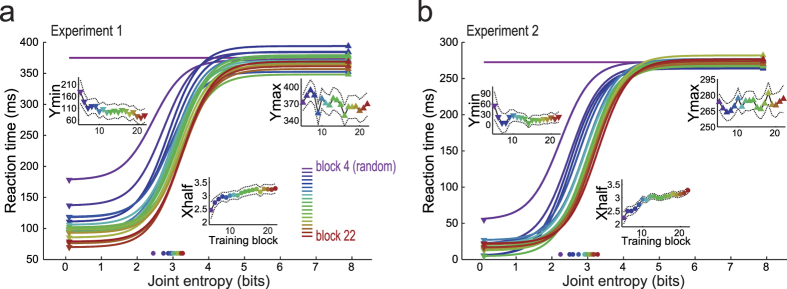
Description of RTs across training blocks by joint entropy following sigmoid curve. A sigmoid function was fitted to the distribution joint entropy values and median RTs for each block from 5 to 22, across all volunteers of Experiment 1 (**a**) or all sessions of Experiment 2 (**b**), respectively. The purple horizontal line represents the mean of median RTs of block 4, the last block of random sequences before the varying sequences. Inset panels show the estimated values (±standard error) of these three parameters along the training blocks. The increase of “Xhalf” values across training blocks is associated to the progressive acquisition of the sequential structure. Similarly, the reduction of “Ymin” is associated to the progressive automation of very simple sequences. The subtle or absent reduction of “Ymax” values indicates that the stimulus-action mapping was (mostly) learned before the blocks expressed on the analysis.

For each training block, we fitted a sigmoid curve relating JE to the median RTs (Fig. 4a,b, for Experiments 1 and 2, respectively). In order to get stable fits, the “slope” parameter had to be fixed to 1, close to the slope of the four parameter sigmoid fitted on analysis of Fig. 3 (not shown). Therefore, the free sigmoid parameters were: (i) “Xhalf” (the JE value which is associated to the middle of the sigmoid curve, which adjusts the leftward/rightward shift), (ii) “Ymax” and (iii) “Ymin” (the RT values observed for sequences of lowest and highest JE). The values of these parameters are presented in the main plot and in the inset plots.

Note the increase of “Xhalf” which is associated to the progressive learning of the sequential structure along training blocks. Note, in addition, the reduction of “Ymin”, associated to the progressive automation of very simple sequences. Finally, in Experiment 1 (Fig. 4a), the “Ymax” had a subtle reduction along blocks (conversely, check the expressive reduction of RTs on random sequence occurred before block 5, in Fig. 1c). In Experiment 2 (Fig. 4b), there were no changes in “Ymax” across blocks: the stimulus-action mapping associated with this parameter value was already optimized in the over-trained volunteer.

## Discussion

This study showed that joint entropy and joint probability are reliable predictors of SRT performance. The relationship between RT and entropy that found in the present study replicates that seen in previous studies^4^,^6^ and, in addition, showed it follows a sigmoid function instead a linear one (Figs 2 and 3). Conjunction of sequence predictability and practice induce major changes in performance. Position and shape of the sigmoid function fit varies systematically across training blocks and function parameters describe training effects (Fig. 4).

This study compared to which extent different predictors explain the variability of RT. Even though these predictors are not psychological models per se, corresponding to mathematical measures of the environment regularity, they may relate to different strategies for accessing environmental regularity aiming at performance modulation. That is, conditional predictors relate to on-line computation, with high cognitive demand, on closed-loop control^13^,^14^. Differently, joint predictors relate to associative strategies^15^ and preparation for chunks of actions, with low cognitive demand, on open-loop control^16^,^17^,^18^. The results of the present study support that open-loop models provide better explanations for sequence learning in SRT. Note that both in random sequences or early trials of a novel sequence there are not learned chunks; thus, in these conditions open-loop control is not applicable and the closed-loop mode drives performance.

Additionally, we compared different functions for relating predictors to RTs. The linear function was an obvious choice, since it is stated by Hick’s Law (“RT increases linearly to the uncertainty of the stimulus”), which was already applied in a variety of contexts, including SRT task studies^6^. The sigmoid function was the other choice, since it describes general psychometric relationships^19^. The sigmoid function explained RT variability better than the linear function. Moreover, the sigmoid curve is observed directly from the data (Fig. 3c, bottom panel). The sigmoid function reflects the high asymptote, the dynamic range and the low asymptote, which may be interpreted as different processes or modes (see 15). That is, the high RT asymptote is associated to untrained or very unpredictable sequences and refers to the reaction mode in closed-loop control, as we described on previous paragraph. The low RT asymptote is associated to very simple sequences extensively trained and refers to full chunking mode in open-loop control. Finally, the dynamic range of the sigmoid function refers to associative or partial chunking mode in sequences which require some closed-loop control. Note that the linear function is restricted to an approximation on the central portion of the dynamic range of the sigmoid curve. Note, in addition, that it is a hard task to define the limits of this portion. Finally, the misuse of the linear function may induce inadequate understanding of the behavioral and physiological outcomes.

The variability of the performance is extensively described by the statistical structure of the sequence. However, one has to consider that aspects of the sequence ignored by our probability and entropy calculations that also influence performance. For example (1), the reversal transitions (or back-and-forth transitions) are performed with lower RTs than the no-reversal transitions^20^,^21^, and (2) within-hands and between-hands transitions are performed in different RTs^22^. The wide set of sequences employed in Experiment 2 allowed investigation of these effects and description of their influence on RT (see Supplementary Fig. S1). However, predictability in patterns which are not expressed in transitions, as the triads of Koch^23^, could be incorporated in an auxiliary set for probability calculations. It seems likely that all regularities on the sequence structure is expressible in terms of probability and entropy.

The present findings support one of the main principles of the Hick’s law, that is, reaction time increases as a function of the amount of information required to make a response. However, they conflict with the notion that this relationship is linear, since data are better described by a sigmoid function at least for the SRT task employed in the study. Furthermore, our results lend support to the view that open-loop processes guide performance of learned sequences and that the joint entropy predictor with sigmoid function provides a satisfactory description of SRT performance across a wide range of conditions. Thereby, this approach provides valuable parameters for studies in psychology and neuroscience of sequence learning.

## Methods

### Volunteers

The Experiment 1 involved 48 healthy undergraduate and graduate students of the University of São Paulo (23 men, 25 women; mean age ±SD = 23.7 ± 2.7, range 18–30) with no previous experience in the SRT task. The volunteers had normal or corrected vision, were right-handed and were not under effects of psychoactive drugs. These volunteers were randomly ascribed to one out of six groups (N = 8 per group), each of them subjected to SRT training with a distinct sequence specifically planned to have critical values of predictability (see Fig. 1B and below).

The Experiment 2 involved a single 26-year-old male graduate student, with extensive prior training in the SRT task involving random sequences of stimuli (see below).

The Ethics Committee of the Biosciences Institute at the University of São Paulo approved the present protocol (CEP-IBUSP: 063/2007). The methods were carried out in accordance with approved guidelines and informed consent was obtained from all subjects.

### Procedure

The SRT task was conducted in a room which luminance was approximately 50 lux at the volunteers’ eyes level, and with attenuated noise. A session lasted less than 30 minutes and was run using a standard personal computer with a custom made MEL (PST) routine.

The volunteer individually sat in front of a computer screen with the eyes positioned at about 60 cm from the monitor where four horizontal lines (50 × 2 mm thick, horizontally spaced by 15 mm), named “a”, “b”, “c” and “d” locations from the left to the right, indicated four possible locations where a single target stimuli, an “X” (40 × 35 mm), could appear. Two mouse devices adapted for allowing the use of both the left and right keys were employed for detecting the responses, one for each hand. Before training the volunteers were instructed to press the mouse button corresponding to the location where the “X” was presented on the screen, using the middle and the index fingers of the left hand for the 1st and 2nd locations, respectively, and the index and middle fingers of the right hand for the 3rd and 4th locations, respectively (Fig. 1a). Upon the correct button press and release the “X” disappeared and a 20 ms inter-trial interval occurred. When the pressed button did not correspond to the actual stimulus location, or when the response was not given until 3 seconds after the stimulus onset, a 50-ms beep was played on a headphone thus indicating the error, and the “X” stimulus reappeared in the same location. Each trial began immediately after the inter-trial interval by the presentation of the “X” either on the same or on another location. The RT corresponded to the time interval between the beginning of the stimulus presentation and the button press.

A complete training session consisted of 1225 trials distributed in 25 blocks, each of them containing 49 trials. At the end of each block the volunteer’s mean RT in that block was printed on the screen along 3 s. On blocks 1 to 4 and 23 to 25 of Experiment 1 all groups were subjected to training using sequences of locations that were random, with the only restriction that one specific location could not be repeated in the next trial (see Fig. 1b, “random sequence”). On blocks 5 to 22 each group was subjected to training using a distinct sequence of locations (see Fig. 1b). The predictability of the sequences of locations varied among groups including the “random sequence”, the “complex probabilistic”, the “simple probabilistic”, the “complex repetitive”, the “simple repetitive”, and the “very simple repetitive”.

In Experiment 2 a single volunteer was subjected to training along 48 sessions, one session per day. Similarly to Experiment 1, on blocks 1 to 4 and 23 to 25 of each session the volunteer was trained using the “random sequence”. In contrast, on blocks 5 to 22, a different sequence was selected for each session, thus variable degrees of predictability were used in this experiment (see Supplementary Table S1 for details).

Figures 1c,d show the RTs of each group/session across the blocks of training. While RTs on blocks 1 to 4 (random sequence for all groups/sessions) were not expected to differ among groups, RTs on blocks 5 to 22 were expected to reflect the impact of predictability of each type of sequence on performance. Finally, RTs on blocks 23 to 25 (random sequence for all groups/sessions) were expected to be influenced by the prior history of training.

### Probability and entropy

The structure of the sequence of locations was described in terms of both the probability theory and the information theory taking into account the existence of four possible locations (“a”, “b”, “c” and “d”), and their relationships with each other. These locations occurring associated (one next to the other) create sets of transitions, composed by two or more locations.

In probability theory applied to sequences, the joint probability (JP) of one transition corresponds to the frequency of that specific transition divided by the frequency of all possible transitions, while the conditional probability (CP) of one transition correspond to the frequency of that transition divided by frequency of subsample of transitions with the same previous locations. Calculation of the probability involved the steps and equations summarized in Fig. 2a. The example of repetitive sequence “a-b-a-b-c-d-c-d” exhibits the circular chain of transitions constituted of only one previous location (“ab”, “ba”, “ab”, “bc”, “cd”, “dc”, “cd” and “da”), which consists of the set of transitions ab, ba, bc, cd, da, dc with the straightforward JPs [2/8, 1/8, 1/8, 2/8, 1/8, 1/8]. The CP of the transition “ba”, referred as the probability of location “a” given the location “b”, *p*(*a*|*b*), is the probability of the transition “ba” (1/8), divided by the frequency of transitions composed by the previous location “b” (i.e. the subset ba, bc, with probabilities [1/8, 1/8], which sums 1/4); so, p(*a*|*b*) = (1/8)/(1/4) = 1/2. Thus, the six-transition set of this example of sequence exhibits the CPs [1, 1/2, 1/2, 1, 1/2, 1/2].

In information theory applied to sequences, joint entropy (JE) is a measure of uncertainty associated with the occurrence of a set of transitions, while conditional entropy (CE) is a measure of uncertainty of the occurrence of the transitions given that the previous locations are known. The calculation of the JE and CE is direct if the probability were previously calculated, using the equations presented in Fig. 3a. The JE is the average across transitions of the log of its JPs weighted by its JPs. The CE is the average across transitions of the log of its CPs weighted by its JPs.

### Data analysis

Analyses were run using built-in and custom made MATLAB (MathWorks) routines. The RT scores were compared using repeated measures analysis of variance (ANOVA) followed by Bonferroni’s post hoc (BPH) test (STATISTICA, StatSoft, Inc.) having Group as the between-subjects factor, and Blocks (1 to 4, 5 to 22, or 23 to 25, for three independent analysis) as a within-subjects factor in Experiment 1. Additional analysis, applied to both Experiments, involved data fitting for linear and sigmoid (equation described by 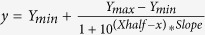) functions using least squares method, which minimizes the sum of squared residuals (R-squared). These functions relate RTs (expressed as raw value of each trial or as median of those) to predictors (JP or CP and JE or CE). Relevant details of the analyses were presented along with the description of the figures. Raw data and analysis scripts are available upon request.

## Additional Information

**How to cite this article**: Pavão, R. *et al*. On Sequence Learning Models: Open-loop Control Not Strictly Guided by Hick's Law. *Sci. Rep.* **6**, 23018; doi: 10.1038/srep23018 (2016).

## Supplementary Material

Supplementary Information
