# Supplementary material for: On Sequence Learning Models: Open-loop Control Not Strictly Guided by Hick’s Law
Source: Sci Rep. 2016 Mar 15;6:23018. doi: 10.1038/srep23018 (PMC4792158; doi:10.1038/srep23018)
Supplement: Supplementary Information [file srep23018-s1.pdf]

SUPPLEMENTARY INFORMATION

On Sequence Learning Models: Open-loop Control Not Strictly Guided by Hick's Law

Rodrigo Pavão, Joice P. Savietto, João R. Sato, Gilberto F. Xavier and André F. Helene

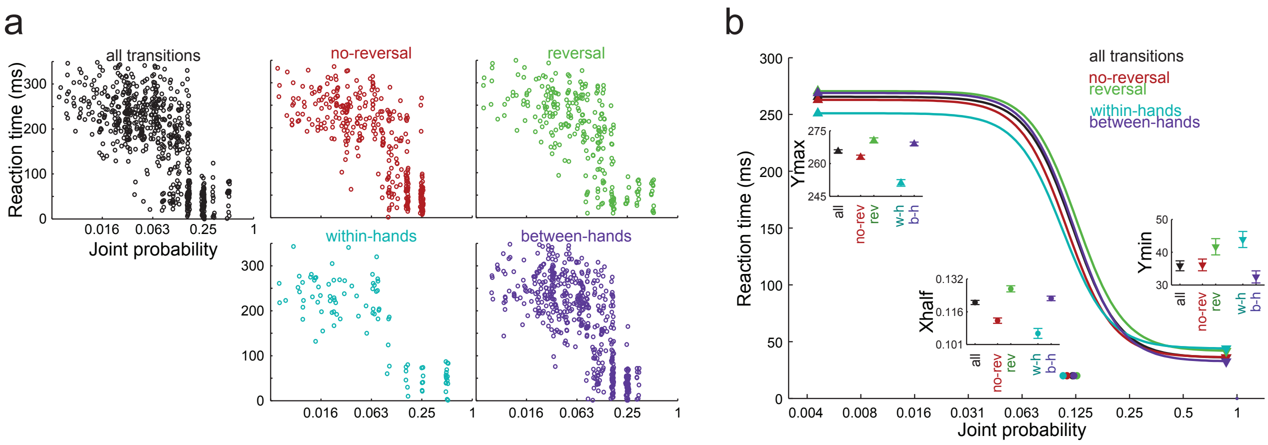

Figure S1. Reaction times vs. joint probability for the reversal, no-reversal, within-hands and between-hands subset of transitions (Experiment 2). (a) RT as a function of joint probability for all transitions (same as Fig. 2d, bottom panel) and for subsets consisting only of no-reversal or reversal transitions, and within-hands or between-hands transitions. Notice that the global relationship described for all transitions was observed on the four subsets of transitions, despite the differences in the density of trials. **(b)** Sigmoid function fitted to the data from all transitions and the four transition subsets. The reversal transitions fit was slightly shifted up and left in comparison to the no-reversal transitions. It replicates previous findings of higher RTs on reversal (Reed, 1994; Vaquero, 2006). Additionally, it also indicates that the access to sequence structure is worse in reversal compared to no-reversal, which was supported by the estimated parameters ($\pm$ standard error) (inset). The between-hands transitions fit is shifted left in comparison to the within-hands transitions. Additionally, as described by the estimated parameters, the between-hands transitions are performed in lower RTs that within-hands in high probabilities; and, inversely, in higher RTs in low probabilities. Similar multifaceted effects on within/between-hands transitions are described in Alain (1993).

Table S1. Sequences performed across the 48 sessions of Experiment 2. Different notations were used for repetitive and probabilistic sequences. For repetitive sequences, the string of letters indicates the repetitive sequence. For probabilistic sequences, the letters indicate the prior locations (one to four letters) and the following four numbers indicate the chance of each location "a", "b", "c" and "d", respectively. The random sequence was performed in session 6: from location "a", the chance of "b", "c" and "d" are equal, from location "b", the chance of "a", "c" and "d" are equal, and so on. The order of the transitions indicates the number of previous locations.

session	sequence	session	sequence
1	dacb	25	a0011 b0011 c1100 d1100
2	(A)	26	cbcbdb
3	dbcacbdcba	27	a0014 b0041 c1400 d4100
4	cbadcabdadcdbca	28	bcad
5	ba	29	a0113 b1031 c1301 d3110
6	a0111 b1011 c1101 d1110	30	a0302 b3020 c0203 d2030
7	cbda	31	a0311 b3011 c1103 d1130
8	acdabdbcab	32	a0112 b1021 c2101 d1210
9	cbdabd	33	a0111 b1011 c1101 d1110
10	dcacab	34	dbdacadbdacbdacacb
11	bacd	35	bdbdbdbdadbd
12	(B)	36	adbd
13	a0013 b0031 c1300 d3100	37	dadbcacb
14	(C)	38	dacbdbcadbcadb
15	(D)	39	a0203 b2030 c0302 d3020
16	(E)	40	a0043 b0034 c3400 d4300
17	(B)	41	bdbcacbcbdad
18	a0013 b0031 c1300 d3100	42	dacbdb
19	(C)	43	a0014 b0041 c1400 d4100
20	(D)	44	a0141 b1014 c4101 d1410
21	(E)	45	a0012 b0021 c1200 d2100
22	a0211 b2011 c1102 d1120	46	dacadadac
23	bdadbcadbcbc	47	bcbdbdacbcadbdad
24	a0411 b4011 c1104 d1140	48	a0031 b0013 c3100 d1300

(A) cabdabdacadbcbdbabdbcacdcbcdacabadadcdcb; (B) 0th order, p(a)=p(d)=0.75, p(b)=p(c)=0.25; (C) 2nd order, ca0013 da0031 cb0031 db0013 ac3100 bc1300 ad1300 bd3100; (D) 3rd order, aca0031 bca0013 ada0031 bda0013 acb0031 bcb0013 adb0031 bdb0013 cac3100 dac1300 cbc3100 dbc1300 cad3100 dad1300 cbd3100 dbd1300; (E) 4th order, caca0031 daca0013 cbca0031 dbca0013 cada0031 dada0013 cbda0031 dbda0013 cacb0031 dacb0013 cbcb0031 dbcb0013 cadb0031 dadb0013 cbdb0031 dbdb0013 acac1300 bcac3100 adac1300 bdac3100 acbc1300 bcbc3100 adbc1300 bdbc3100 acad1300 bcad3100 adad1300 bdad3100 acbd1300 bcbd3100 adbd1300 bdbd3100.
